# Transformation of acute cholecystitis to acute choledocholithiasis in COVID-19 patient

**DOI:** 10.1016/j.amsu.2021.102946

**Published:** 2021-10-14

**Authors:** David Song, Harinivaas Shanmugavel Geetha, Andrew Kim, Tasur Seen, Talal Almas, Vikneswaran Raj Nagarajan, Noor Alsaeed, Jui Hsin Cheng, Joseph Lieber

**Affiliations:** a Icahn School of Medicine at Mount Sinai - Elmhurst Hospital Center, Elmhurst, NY, USA; b Saint Vincent Hospital, Worcester, MA, USA; c New York Institute of Technology College of Osteopathic Medicine, Westbury, NY, USA; d RCSI University of Medicine and Health Sciences, Dublin, Ireland

**Keywords:** Coronavirus 2019, COVID-19, Cholecystitis, Cholidocolithais, SARS-CoV-2

## Abstract

The global pandemic of Coronavirus 2019 (COVID-19) or SARS-CoV-2 has numerous manifestations in different organ systems. It is known that SARS-CoV-2 infects the hepatobiliary system leading to presentations such as acute cholecystitis, choledocholithiasis and hepatitis. Although the exact mechanism of the underlying pathology is unknown, it is likely attributed by the tropism of the virus to the ACE2 receptors in the hepatocytes and bile duct cells resulting in a cytokine storm that precipitates as systemic symptoms from acute COVID-19 infection. In this case report we present a case of a 47-year-old male who presented with signs consistent with acute cholecystitis. It was confirmed on ultrasound and he was incidentally found to be positive for COVID-19 on routine surveillance testing. He was asymptomatic and was being prepped for cholecystectomy, but developed an acute elevation of liver enzymes suggesting choledocholithiasis. After endoscopic retrograde cholangiopancreatography (ERCP) and cholecystectomy the patient experienced a rapid normalization of liver enzymes and improvement of his abdominal symptoms.

## Highlights

• SARS-CoV-2 can infiltrate gallbladder wall cells, mimicking acute cholecystitis and choledocholithiasis.
• Exact mechanism is unknown, but it is likely attributed by the tropism of the virus to the ACE2 receptors in the hepatocytes and bile duct cells resulting in a cytokine storm that precipitates as systemic symptoms from acute COVID-19 infection.
• Patients with acute cholecystitis and COVID-19 require close monitoring to prevent the progression into cholidocolithasis and cholangitis.

## 1 Introduction

Stones in the hepatobiliary system are causative of various hepatobiliary disease presentations. It is estimated that 6.3 million men and 14.2 million women between the ages 20–74 have gallbladder disease, the main presentation being acute cholecystitis which is predominantly caused by gallstones [1]. Within the general population with symptomatic gallstones, 10% will also have choledocholithiasis which increases to 15% when patients are presenting with acute cholecystitis [2]. Symptomatic acute cholecystitis includes fever, right upper quadrant (RUQ) abdominal pain, positive Murphy's sign and in some cases jaundice. Symptomatic choledocholithiasis includes colicky RUQ abdominal pain with right shoulder radiation, intermittent jaundice accompanied by pale stools and dark urine. The complications of cholecystitis and choledocholithiasis include gallbladder perforation, biliary obstruction, pericholecystic abscess, fistula, bacterial cholangitis, and pancreatitis [3].

COVID-19 is known to enter host cells by binding to the ACE-2 receptors, which are commonly found throughout various organs including the liver, gallbladder and in high levels in the bile duct [3]. Furthermore, it has been discovered that SARS-CoV-2 can infiltrate gallbladder wall cells, mimicking acute cholecystitis [3]. Previous studies have shown that acute calculous cholecystitis can be responsible for progression into concurrent choledocholithiasis [4]. However, what is not well understood is the pathogenesis of SARS-CoV-2 in cholecystitis and whether it can be responsible for the progression of cholecystitis to choledocholithiasis. We hereby delineate an interesting case of a patient who had an acute transformation of cholecystitis to choledocholithiasis with concurrent asymptomatic COVID-19 infection. Further workup reaffirmed an ongoing COVID-19 infection. Notably, this work has been reported in accordance with the SCARE guidelines [5].

## 2 Case presentation

Patient is a 47-year-old Hispanic male with a past medical history significant for hypertension, hyperlipidemia and non-insulin dependent diabetes mellitus presented with 4 days of non-radiating RUQ abdominal pain worsening with fatty meals. His symptom started abruptly with associated nausea and non-bloody vomiting. In the emergency department RUQ ultrasound was performed which was significant for gallbladder sludge and the presence of a 2.3 cm stone in the gallbladder neck consistent with acute cholecystitis without dilated common bile duct (CBD). General surgery was consulted with a plan for non-emergent cholecystectomy. The patient was subsequently started on broad-spectrum antibiotics coverage with ceftriaxone 1 g every 24 h and metronidazole five hundred milligrams every 8 h along with intravenous (IV) fluid administration. He was incidentally found to be COVID-19 positive on routine surveillance testing, but did not have any respiratory distress such as shortness of breath, pleuritic chest pain or changes in his exercise tolerance. Furthermore, he did not require any supplemental oxygen and previously received two doses of the Moderna vaccine. As the patient was asymptomatic, COVID-19 treatment (which included dexamethasone, remdesivir, and tocilizumab) was not initiated.

On admission labs were notable for alanine transaminase (ALT): 58 U/L aspartate aminotransferase (AST): 62 U/L, total bilirubin: 0.6 mg/dL, direct bilirubin: 0.2 mg/dL, and C-Reactive Protein (CRP): 182.3 mg/L. Other labs including basic metabolic panel, complete blood count, lipase, coagulation panels and blood cultures were all unremarkable. Pertinently, the remaining battery of blood tests, including full blood count (FBC), alkaline phosphatase, and cardiopulmonary tests were unremarkable. DS admitted the patient and referred the patient for further consultation and workup.

**Fig. 1:**
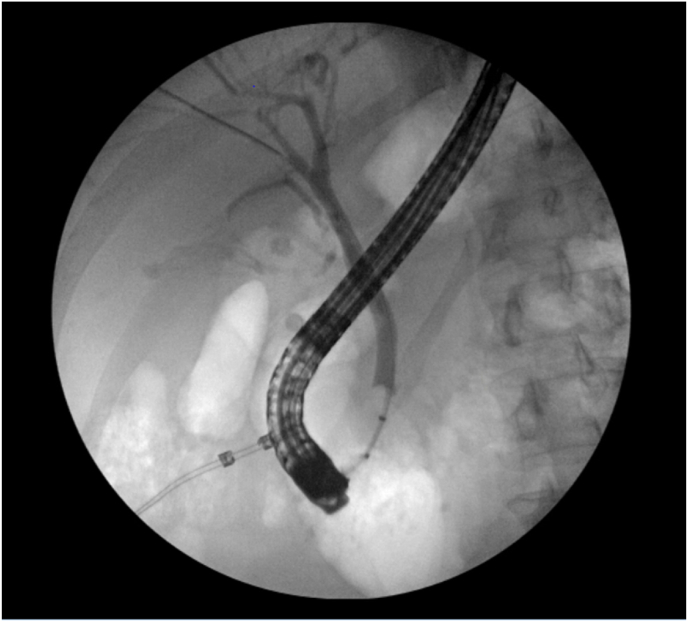
ECRP showing dilated CBD with presence of sludge without evidence of stone.

On the day of surgery, the patient's ALT and AST were elevated to 583 and 944 respectively. Total bilirubin and direct bilirubin were elevated to 2.9 mg/dL and 2.7 mg/dL respectively. These suggested the advancement of the stone into the CBD and resultant choledocholithiasis. The patient denied worsening of his abdominal pain during this time. Gastroenterology was consulted for acute choledocholithiasis. Given the acute elevation in liver enzymes without fever, hemodynamic instability and leukocytosis, the diagnosis of choledocholithiasis without cholangitis was made and ERCP was performed to relieve biliary obstruction. ERCP revealed dilated CBD with evidence of biliary sludge, but without evidence of stones (Fig. 1). The histopathological examination of the gallbladder was not conducted, and the eventual diagnosis was based predominantly on the slate of laboratory workup results, physical examination and ultrasound findings. *Helicobacter pylori* testing was also negative at this time. Patient's lab values showed improvement post-ERCP (Table 1) and cholecystectomy was performed. Imperatively, the patient did not manifest any additional COVID-related symptoms, and a general review of systems, including neurological examination, was unremarkable. There were no mental status changes, focal neurological deficits, numbness or tingling noted. Furthermore, computed tomography (CT) scan revealed no appreciable abnormality. Patient was followed up as an outpatient post-surgery with improvement of his abdominal symptoms and his lab values normalized.

**Table 1:** Lab values showing the conversion of acute cholecystitis to acute choledocholithiasis.

Lab values	Day 1 (On admission)	Day 2	Post- ERCP
**ALT (U/L)**	58	583	260
**AST (U/L)**	62	944	69
**Total Bilirubin (mg/dL)**	0.6	2.9	1.4
**Direct Bilirubin (mg/dL)**	0.2	2.7	0.9

## 3 Discussion

SARS-CoV-2, the pathogen responsible for COVID-19, has caused morbidity and mortality at an unprecedented scale globally [6]. The acute and subacute effects of COVID-19 infection are still being studied with the disease process showing a wide array of clinical manifestations in different organ systems including the hepatobiliary system.

COVID-19 infection can present with a variety of gastrointestinal symptoms, but sometimes with the absence of respiratory symptoms as in our patient. These clinical manifestations can be explained by an independent tropism of the virus for hepatic and bile duct cells [7]. SARS-CoV-2 is characterized by the presence of a viral protein spike that interacts with the ACE2 receptor in the host and this receptor is broadly expressed in the liver, gallbladder and bile ducts [3]. This suggests that COVID-19 can localize and directly affect the gallbladder and bile ducts that may induce contraction.

SARS-CoV-2 also infects hepatocytes which was proven by autopsies of COVID-19 patients demonstrating liver tissue with different patterns of hepatic injury and viral RNA was also found inside the hepatocytes [7]. In the biliary apparatus several instances of COVID-19 infection leading to acute acalculous cholecystitis have been reported. As the number of cases reporting COVID-19 infection associated with biliary inflammation grows, there is an increased need to understand the pathogenesis underlying it. One major mechanism that explains the high incidence of cholecystitis and choledocholithiasis in COVID-19 patients is direct infection. Xu et al. reported that cytolysis with mild transaminitis has been observed in patients with SARS-CoV-2 suggesting that the virus causes direct injury to the bile duct cells, thereby causing acute inflammation of the gallbladder and bile duct [8].

Ying et al. described the reverse transcription polymerase chain reaction (RT-PCR) of the bile after percutaneous drainage of gallbladder in a patient with sludge and acute cholecystitis did not detect SARS-CoV-2 [9]. This negative RT PCR could be explained by a complication of COVID-19 infection such as a profound cytokine storm that has also been demonstrated in relapsing multiple sclerosis patients receiving alemtuzumab who developed acute cholecystitis following a cytokine storm [7,10].

COVID-19 infection also increases the incidence of systemic endotheliitis, hypercoagulability, APLA and thrombotic microangiopathy which collectively contribute to the occurrence of cholecystitis [6]. Studies have demonstrated that COVID-19 infection upregulates the expression of proinflammatory cytokines such as interleukin-6 and tumor necrosis factor-alpha that trigger a cytokine storm which recruits macrophages and causes inflammatory reactions similar to those of vasculitis and activation of thrombophilic status [11]. This hypercoagulable state induced by COVID-19 can lead to gangrenous cholecystitis arising from vascular insufficiency and it evolves into necrosis and perforation of the gallbladder wall with bile leakage. This is evidenced by the poor clinical outcomes and mortality rates experienced by COVID-19 patients undergoing surgery for cholecystitis around the world prompting changes in several surgical societies as suggested in the Tokyo guidelines recommending delaying surgical operation and to perform percutaneous gallbladder drainage for high risk patients [10]. A similar mechanism has also been described in viruses such as Human immunodeficiency virus (HIV) and hepatitis B which have been implicated in acute acalculous cholecystitis. Similarly, COVID-19 has been associated with vascular inflammation that could potentially be leading to ischemic gallbladder changes independent of the development of shock [12]. Additionally, COVID-19-related coagulopathies and thrombosis could also have triggered the patient presentation; however, given that the ultrasound imaging did not divulge any such information, this etiology was considered less likely. As we continue to learn of the various new manifestations of COVID-19 and considering the global scale of this pandemic, it is clear that there will be a great need to understand the underlying pathogenesis and complications arising from COVID-19 infection which will better equip us in managing such complications.

In recent times, several reports have also highlighted gangrenous cholecystitis as a potential cause of vascular insufficiency in patients afflicted with COVID-19 [13]. Therefore, vascular insufficiency might indeed be the causative factor in the present patient, heralding the transformation of acute cholecystitis to choledocholithiasis. A debilitating COVID-19 infection can lead to damage of the hepatobiliary system, thereby leading to presentations such as acute acalculous cholecystitis, choledocholithiasis and hepatitis. Patients with acute cholecystitis and COVID-19 therefore require close monitoring to prevent progression to choledocholithiasis and cholangitis.

## 4 Conclusions

As physicians continue to be perplexed by the myriad of manifestation of COVID-19, it is crucial to remain cognizant of progression from cholecystitis to choledocholithiasis. Close monitoring in COVID-19 patients with signs and symptoms typical of acute cholecystitis is therefore crucial in order to thwart the aforesaid transformation.

## 5 Limitations

There are limited cases and literature on the hepatobiliary system and the involvement of COVID-19. Our case represents only one encounter where an acute COVID-19 infection caused a contraction of the gallbladder induced by ACE2 receptors transforming acute cholecystitis to acute choledocholithiasis. Furthermore, within our case, histopathological workup results were unavailable, further compounding the diagnostic conundrum encountered.

## Disclosure

None.

## Author contribution

DS wrote the abstract, case presentation, study concept, design, conclusion, limitation.

AK wrote the introduction.

TS, VRN, NA, JHC: revised the edits, figures, chart review.

HSG wrote the discussion, data collection.

DS, AK, TS, TA, JL performed the final edit.

## Funding

None.

## Ethical approval

Obtained.

## Consent

Obtained.

## Registration of research studies

Name of the registry: NA.

Unique Identifying number or registration ID: NA.

Hyperlink to your specific registration (must be publicly accessible and will be checked): NA.

## Guarantor

Talal Almas.

## Declaration of competing interest

None.
